# A phenomenological approach to the phenomena of anxiety: a scoping review

**DOI:** 10.3389/fpsyg.2026.1921674

**Published:** 2026-10-06

**Authors:** Eduardo Silva, Willyan da Costa Mota, Camila Souza, Lucas Guimarães Bloc

**Affiliations:** Post-Graduation Program in Psychology, University of Fortaleza, Fortaleza, Brazil

**Keywords:** anxiety disorders, clinical phenomenology, obsessive - compulsive disorder, phenomenological psychopathology, phenomenology, scoping review, social anxiety

## Abstract

The increasing number of diagnoses of anxiety disorders has highlighted the need for discussions about anxiety in its affective, psychopathological, and mundane dimensions. This scoping review mapped 36 scientific articles (2014–2025) to analyze how Phenomenology contributes to the understanding of anxiety as a global phenomenon. The results highlight trends in the field in 4 discursive categories. For authors of Phenomenological Psychopathology, anxiety is understood as a global alteration of being-in-the-world, characterized by disturbances in temporality (dysrhythmia, narrowing of the future), spatiality (feeling of invasion/avoidance), and intersubjectivity (hypervigilance towards others). In specific conditions such as social anxiety and OCD, anxiety appears as a way for the subject to deal with anguish and uncertainty. In phenomenological philosophy, anxiety (or anguish) is considered a human and existential phenomenon, enabling a confrontation with incompleteness and freedom. The clinical directions of the collected articles point to the ways in which clinical contact can, by enabling contact with uncertainty, pave the way for a creative production of meaning. The review concludes that, although the articles start from different focuses on anxiety, there is an observable tendency in strands of production understanding anxiety as a way of inhabiting the world. However, a gap is identified in the literature regarding the articulation of these phenomenological conceptions with the specific sociocultural contexts of contemporary times.

## 1 Introduction

The significant increase in complaints of anxiety in the general population has brought to the forefront the discussion about anxiety disorders (Javaid et al., 2023). It is estimated that, on average, 4% of the global population currently has an anxiety disorder, totaling approximately 359 million people worldwide, making these disorders the most common of all mental disorders (World Health Organization, 2025).

Nosologically, the term “anxiety” is understood as an affective state, and can refer both to the set of symptoms related to it and to a diagnostic category of mental disorders (Frota et al., 2022; D’ávila et al., 2020). As a set of symptoms, it can be triggered by vague feelings of fear, tension, and anguish, as well as by physiological manifestations of tachycardia, muscle tension, and sweating, amongst others (Javaid et al., 2023; Dalgalarrondo, 2019; Rós et al., 2020). As a diagnostic category, anxiety disorders can be understood as disturbances related to experiences and behaviors governed by anxiety and fear, provided that these are not understood as adaptive responses to the situations in which they manifest (American Psychiatric Association, 2023).

Anxiety can manifest as a specific disorder, organized in the DSM-5 into nine diagnostic categories: separation anxiety disorder, selective mutism, specific phobia, social anxiety disorder (social phobia), panic disorder, agoraphobia, generalized anxiety disorder (GAD), substance/medication-induced anxiety disorder, and anxiety disorder due to another medical condition. What distinguishes these conditions from each other is the type of object or situation that triggers fear, anxiety, and avoidance behaviors, considered central symptoms of the clinical presentations described (American Psychiatric Association, 2023). However, beyond this categorical organization, it is noteworthy that anxiety also presents itself as a comorbid phenomenon/symptom in several psychopathological conditions, such as bipolar mood disorder (Kinrys et al., 2019), obsessive-compulsive disorder (OCD) (Mangolini et al., 2019), schizophrenia (Buonocore et al., 2018), and depressive disorders, in which it has a high prevalence (Mangolini et al., 2019).

With regard to OCD, it should be noted that, with the change from DSM-IV to DSM-V, this disorder was removed from the group of anxiety disorders and relocated to the category of obsessive-compulsive and related disorders (American Psychiatric Association, 2023). Although this change seeks to distinguish conditions with their own dynamics, OCD remains strongly influenced by manifestations of anxiety, which points to possible limitations of nosology in capturing the complexity of the anxious experience and reinforces the need for approaches that consider its plurality beyond the diagnostic boundaries based on the biomedical model (de Araújo, 2022). Furthermore, since this is a relatively recent reorganization and not yet agreed upon among the different fields of psychopathology, it is possible to find in the literature productions that support its permanence within the field of anxiety disorders (Jalal et al., 2023) as well as works that problematize and criticize this classificatory change (Hlynsson et al., 2025).

Beyond its demarcations in the psychopathological field, sometimes as a specific disorder or as a symptom, anxiety is also a phenomenon situated in everyday life, manifesting itself as an affective state present in the most diverse moments of one’s life (Minkowski, 1966/2000). The polyphony of anxiety reveals that its foundation lies in human existence itself (Micali, 2022), and boundaries present in traditional diagnostic categories point to the need to return to a path that understands it as a global experience present in different ways of existing. It is a phenomenon that involves multiple dimensions - biological, clinical, scientific, and philosophical- interconnected with each other, reinforcing its complexity and its impossibility of being understood by a single field of knowledge (Glas, 2013). This plurality, although sometimes fruitful, has hindered the systematic articulation between the contributions of psychopathology, clinical psychology, and existential philosophy, generating a gap concerning the integration of these bodies of knowledge into a cohesive framework that can directly inform clinical practice in mental health.

Within this framework, phenomenological approach to the phenomena of anxiety presents itself as a field of knowledge that, since its origin, prioritizes the understanding of experience in its totality, without reducing it to exclusively causal or objectifying explanations (Souza et al., 2021; da Silva, 2023). By recognizing that all human experience, including that of suffering, is constituted in an inseparable way between biological, clinical, scientific, and philosophical dimensions, phenomenology offers a path of investigation capable of supporting the complexity of the phenomenon of anxiety (Tamelini and Messas, 2022).

As a field of scientific production, however, phenomenological research may operate through distinct levels of analysis of the lived experience: either through the structure of conscience and of the Dasein in existential phenomenology (Hayes and Adams, 2019), through the investigating structural changes of the lived world in phenomenological psychopathology (Bloc et al., 2026), or through adapted qualitative research towards exposing the first person perspective in empirical phenomenological methods (Guedes and Moreira, 2009). These differing analyses produce a seeming tension between philosophical tradition and clinical practice, as they concern themselves with a shared object (the lived experience) but may differ in objective and systems of rigor (Bloc et al., 2026). Without rejecting these tensions, it is a choice to consider that the relationship between these approaches can be harvested towards a constructive synthesis, as clinical research and philosophical tradition refer back to one another in the actualization of new models of experience (Tatossian, 1979/2006).

This synthesis operates through at least three distinguishable models by which the phenomenological scope can inform clinical understanding: (a) as a methodological tool, structuring data collection and analysis in qualitative investigations of anxiety (e.g., through phenomenological interviewing or the construction of validated assessment instruments); (b) as a theoretical basis for the construction of psychopathological concepts, offering the conceptual vocabulary and the clinical understanding (e.g., dysrhythmia, hyperreflexivity); (c) as a conceptual reference for therapeutic intervention, informing the clinician’s stance and the qualitative attention given to the patient’s lived experience within the therapeutic encounter (e.g., clinical cases). Thus, considering this diversity, an understanding of the phenomenological approach to anxiety as a field of scientific production is considered necessary. Here, the term “phenomenological approach” is used to indicate a methodological and conceptual scope that aims to explore the lived structures of anxiety through articulations with phenomenological research in the range of disciplines mentioned above. Given the different ways of understanding anxiety, as well as the increase in diagnoses in recent years, the following question arises: how has the scientific literature discussed anxiety through phenomenology? In order to answer this question, this article aims to map, describe, and synthesize how the scientific literature has addressed anxiety in the field of phenomenology, identifying conceptual trends, theoretical gaps, and emerging research directions through the elaboration of a scoping review.

## 2 Methodology

The following research proposes to elaborate a scoping review based on an original research protocol, grounded in the guidelines proposed by the Manual of Evidence Synthesis (Aromataris and Munn, 2020), published by the Johanna Briggs Institute. The facilitating model that will be used to structure this research comes from the Preferred Reporting Items for Systematic Reviews and Meta-Analyses (PRISMA, 2020) electronic guide, specifically its extension for scoping reviews (PRISMA-ScR; Tricco et al., 2018).

Adapting from these sources, the elaboration of the scoping review will be carried out in seven main consecutive steps: (1) definition of the guiding question and objective of the research; (2) search for relevant sources and databases that enable the purpose of the review; (3) selection of studies according to the criteria defined by the research protocol; (4) data mapping, based on reading the studies and identifying relevant information; (5) synthesis of information and summarization of results; (6) analysis of the synthesized information in order to identify patterns in the literature; and (7) presenting the results, presenting an overview of the literature and identifying possible gaps in the research (Arksey and O’Malley, 2005; Aromataris and Munn, 2020). Thus, as a starting point for this data collection, the following question is posed: how does phenomenological literature investigate the anxiety phenomena today?

### 2.1 Research strategy

For data collection, the scientific portals BVS Psi, Scopus, Web of Science, and PubMed were designated. In the searches conducted, only studies that met the following inclusion criteria were considered: those with a published status; published from 2014 onwards; published in Portuguese and/or English; and responding to the keywords. Bearing in mind the multiplicity of clinical, psychopathological, and contemplative meanings that the term “anxiety” can adopt, the search string used sought to be as expansive as possible. As such, the following formulation was obtained:

(“phenomenology” OR “phenomenological” OR “phenomenological approach” OR “phenomenological analysis” OR “lived experience” OR “existential perspective”) AND (“anxiety” OR “anguish” OR “angst” OR “fear” OR “generalized anxiety disorder” OR “GAD” OR “panic disorder” OR “social anxiety disorder” OR “social phobia” OR “specific phobias” OR “separation anxiety disorder” OR “selective mutism” OR “substance/medication-induced anxiety disorder” OR “anxiety disorder due to a medical condition” OR “obsessive-compulsive disorder” OR “OCD”).

Bearing in mind searching mechanism restrictions across different platforms, variations on this initial string were used and compiled in Appendix 1. Works that address anxiety disorders from a biomedical perspective were effectively excluded from the review. After reading the articles in full, those that did not address anxiety from a phenomenological perspective and that were not related in some way to the implications in the clinical context were excluded. This stage took place between April and June of 2026.

### 2.2 Evidence selection

The articles collected in this first stage were exported to the EndNote Online citation platform (Clarivate, 2024), where all duplicates were excluded. In this way, 17,273 articles were identified (BVS: 2,942; Scopus: 6,477; PubMed: 3,319; Web of Science: 4,535) that matched the descriptors in both languages. From these, after filtering by reading titles and abstracts, 148 articles were kept.

For the second stage of selection, the selected articles were exported to the Rayyan platform (Ouzzani et al., 2016), whose functionalities are dedicated to the production of systematic literature reviews. In this way, three independent reviewers performed a blind reading of the articles, indicating their choices to maintain or remove articles based on the scope and criteria of the review through the platform. After every reviewer had assigned a decision and written justification of their stance to each article, a meeting was held in order to resolve divergences in judgement. The excluded articles in this stage were judged to not respond this research’s driving question, as they fell into the following categories: studies that address anxiety as a sociodemographic factor of its subjects without a phenomenological analysis; studies that rely on medical definitions of anxiety for its discussion; studies that utilize phenomenological methods without addressing phenomenological readings of anxiety. Purely theoretical or philosophical articles were retained when their conceptual argument was understood to be oriented towards informing clinical understanding, diagnostic reasoning, or therapeutic practice as informed by a phenomenological lens, regardless of the absence of empirical patient data or applied phenomenological method (Figure 1).

**Figure 1:**
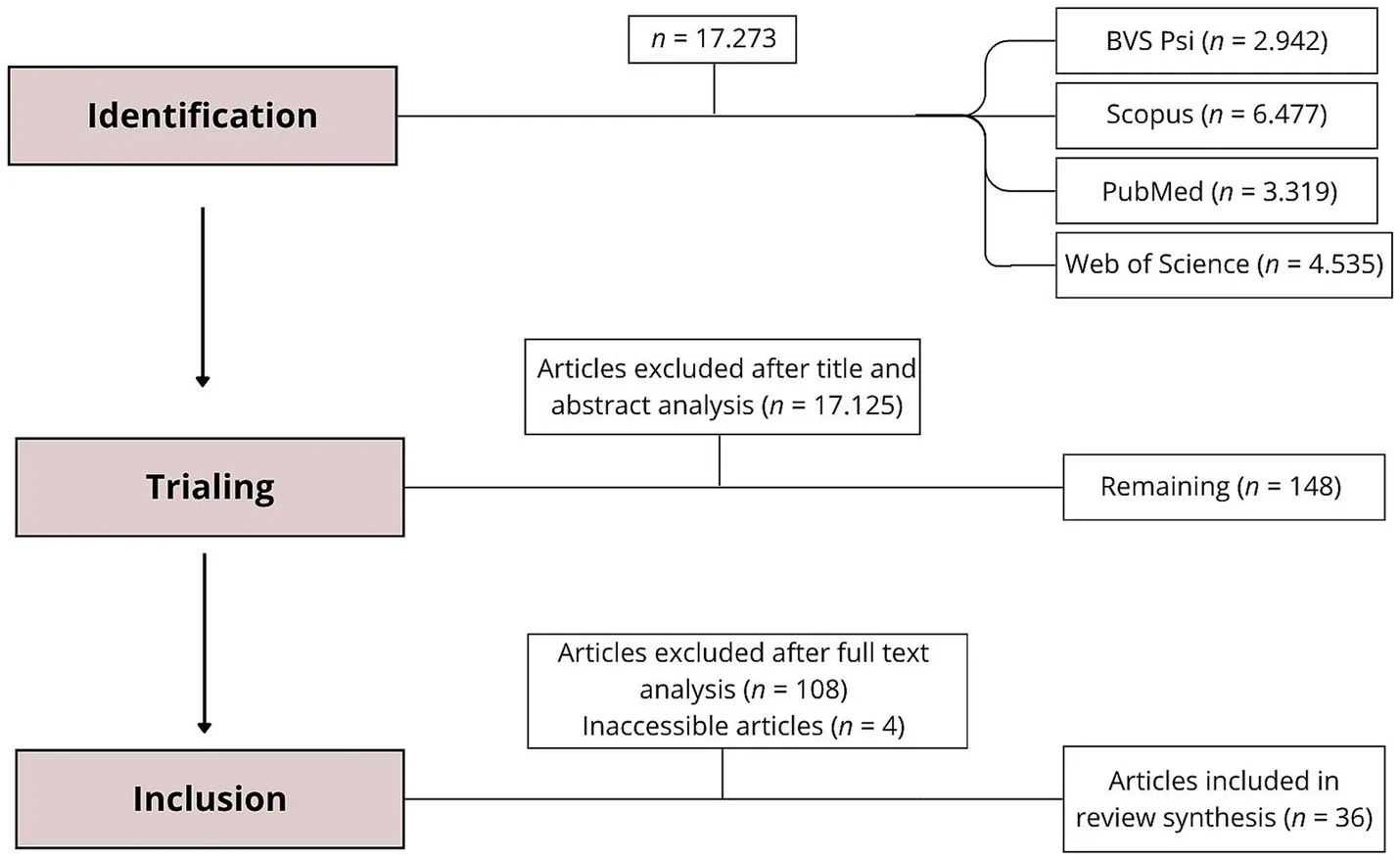
Flowchart for identifying and collecting literature.

In this process, 36 studies were identified as eligible for inclusion, and their respective metadata were grouped for this review. In order to organize the collected content, the following data were highlighted and tabulated: field of study; philosophical/theoretical basis; research method; publication journal; publication period; and authors. The remaining articles were excluded because they did not use a phenomenological lens to look at the phenomenon of anxiety, even though many used phenomenological research methods in their work.

## 3 Results

The data collection reveals a predominance of studies that use a phenomenological approach to conduct research directed towards clinical perspectives on anxiety, mostly concentrated in the fields of Psychology and Psychiatry, represented by 26 articles. These show notable agreement among themselves regarding the lived experience of anxiety, suggesting a common basis of both scientific knowledge and phenomenological investigation. However, several articles have distinct investigative focuses on the anxious experience, so their results were subsequently divided into topics for discussion.

It is also noteworthy that there is a strong representation of productions on anxiety from a phenomenological-existential philosophy perspective, comprising 10 articles. Unlike studies with a clinical focus, which may revisit the philosophical bases that underpin phenomenological clinical practice, this category of articles focuses on the categorical meaning of the anxious experience, keeping in mind the classical and contemporary philosophical literature of phenomenology. The contribution of these articles, considering the scope of this review, lies in the way the concept of anxiety is revisited today, contrasting and bringing it closer to modern scientific literature.

The journals and periodicals, listed in tables below, reveal the prevalence of themes in the articles. The journal Phenomenology and the Cognitive Sciences appears repeatedly, having 7 publications (19.4% of the total), and is directly followed by the journals Psychopathology and Philosophy, Psychiatry & Psychology, with 5 articles each (13.8% each), and then Frontiers in Psychology, with 2 articles (5.5%). The content related to these journals is predominantly focused on clinical discussions about anxiety, representing 53.8% of the articles that make up this category, and 38.7% of the total collection of articles. The other journals, both clinical and philosophical in nature, have 1 publication each (2.7%).

The distribution of authorship of the collected articles seems to represent the breadth of discussions about anxiety. With the exception of the articles by Kristiansen (2023a, 2023b, 2024, 2025a, 2025b) concerning lived experience in cases of social anxiety, and Bradley (2015a, 2015b) with contributions from philosophical phenomenology on anxiety, there are no repetitions of authors, regardless of the field of study.

Furthermore, the distribution of methodologies shows a strong prevalence towards theoretical studies, which make up 27 (or 75%) of the total collection. The remaining studies are qualitative empirical in nature, and mostly utilize phenomenological methods of content analysis alongside a clinical or psychopathological investigation of anxiety, with the exception of the construction and experimental validation of a clinical instrument by Lu et al. (2024). While it is not in the scope of this review to weigh and evaluate the different levels of evidence of these articles, it is sensible to point this tendency as not every conceptual work necessarily has an empirical counterpart; this is justified by a shared inquiry on the phenomenology of anxiety, rather than through methodological equivalence.

The presentation of the collected articles is arranged into two groups of results. In the first group (Anxiety and Clinical Perspectives), studies whose assumptions and results address a clinical and psychological bias regarding anxiety were grouped. These were subsequently subdivided into topics: productions from the field of Phenomenological Psychopathology (Alterations of structure in anxiety); productions that discuss anxiety as a central axis of specific psychopathological conditions (Anxiety in specific configurations: OCD and Social Anxiety); and theoretical-practical productions that use Phenomenology as an interventional lens in the clinic (Clinical practices). The second group (Anxiety and Existential Perspectives) is composed of articles that, for the most part, revisit philosophical notions about concepts such as “anguish” and “fear,” but which have a proximity to phenomenological notions of anxiety. The following studies were divided into their respective groups and subgroups of results, accompanied by numbered tables.

### 3.1 Anxiety and clinical perspectives

#### 3.1.1 Changes in the structure of experience in anxiety

The starting point for a phenomenological understanding of anxiety lies in understanding first-person experience. In the field of Phenomenological Psychopathology, it is understood that different forms of illness show their impacts on the structures that underpin subjectivity, altering the ways in which an individual experiences their own time, space, body, intersubjectivity, and sense of self. In this sense, articles 1, 7, 14, 16, 17, 18, 19, 23, 24, and 25 seek to unveil the possible alterations of experience present in the anxious experience.

For the most part, the collected literature points to an altered relationship with time as one of the possible main axes of the anxious experience. In phenomenological literature, temporality is, in addition to being a sensory experience, relates to the construction of a personal narrative, constituted by the individual in order to take root in the world (de Souza et al., 2020). A description of the perception of time anxiety can be a valuable foundation for understanding how anxious functioning accommodates (or even displaces) a being’s relationship with the world. It is through this approach that studies 1, 14, 17, 16, and 23 seek to describe the anxious experience.

Discussions about anxiety in its affective axis are frequently used by the aforementioned articles, sometimes described as a feeling of constriction or confinement of the senses. The discussion elaborated by article 1, by Aho (2020), is emblematic of this concept. In a theoretical study, the author considers that lived anxiety implies a “vital dysrythmia” through an understanding of lived time as an embodied dimension. Through a collection of first-person accounts in literature, the author attempts to show how anxiety can be seen as an acceleration of both the bodily processes and cognition, in a way that makes self-organization difficult. Furthermore, the discussions of article 17, by Kristiansen (2023b), attempt to elaborate on how these alterations could manifest themselves, bearing in mind the human capacity to project oneself in time as a way of seizing one’s possibilities. Time loses its expansive character in the face of anxiety, so that the perceptual field focuses on a single possibility, typically aversive and rapidly approaching the subject.

Articles 14 and 23, by Holzhey-Kunz (2020) and Moskalewicz and Schwartz (2020) respectively, delve into broader discussions about disturbances in temporality as a common axis in mental illness. These studies converge in their theoretical considerations by positioning a methodological difficulty in objectively discussing temporality in psychopathology and individually postulate possible hermeneutical alternatives to this problem. However, the authors argue that people vulnerable to experiences of disturbance in temporality may also be susceptible to anxious experiences.

Subjectivity is not constituted by just one foundation, in that psychic illness implies the alteration of several axes of experience simultaneously. Thus, although most phenomenological literature focuses on the alteration of experience situated in time, it is not an isolated phenomenon, mutually implying relationships with spatiality, corporeality, and intersubjectivity. It is from this perspective that articles 4, 12, 18, 19, 20, and 22 position their discussions.

Spatiality and anxious alterity are discussed within the framework of social anxiety on articles 4 and 18, by Boyle (2019) and Kristiansen (2024) respectively. In studies that analyze first-person descriptions, the authors seem to have reached an understanding of the anxious world as social relations and geographical space acquire a threatening quality. The experiences of the anxious world and the fear related to it acquires a target: the Other, whether real or not, becomes a threatening figure, capable of alienating an individual from themselves through their judgment. The authors posit that this happens alongside a relationship of unfamiliarity with lived space. The interviewed subjects report feeling like an invader of other people’s space, and at times, they seem to have their space constantly invaded, in a dynamic that Kristiansen (2024) coins “intersubjective domination.” Sharing a similar set of descriptions, Boyle (2019) considers the lived experience of inhabiting threatening spaces and relations through the perspective of habituation, as these experiences disrupt the ability of people with anxiety disorder to unreflexively belong in their own lives and routines. This discussion seems to start from a point similar to the articles previously mentioned, considering anxiety as an affect related to the denaturalization of the world of possibilities, that arouses feelings of anguish and fear without necessarily establishing a causality between them.

Keeping in mind the framework of social anxiety, study 23, by Kristiansen (2023a), situates the lived-body as an axis of experience that is traversed and instrumentalized by the gaze of the Other. The interviewed patients report feeling like they are constantly performing a social performance, and that their bodies are the vehicles for this to happen, so that the only plausible way out seems to be to hide from the sight of others. Furthermore, they also report feeling that they would like to forget about themselves for a while, considering the constant self-evaluation they perform, which may point to a possible clinical direction to be considered.

#### 3.1.2 Anxiety in specific settings: OCD and social anxiety

Although psychopathological discussions about anxiety are evident in the other categories of this article, these focus mainly on understanding anxiety as a specific phenomenon, that is, without direct correlation with other modes of unhealthy existence. Therefore, it is understood that it is necessary to understand how anxiety presents itself in diverse psychopathological conditions. Articles 4, 5, 9, 10, 11, 16, 17, 18, 19, 20, and 21 were grouped together because they present discussions related to specific anxiety disorders. Here, discussions about Social Anxiety Disorder and Obsessive-Compulsive Disorder are highlighted, which have historically been associated with Anxiety Disorders.

Social anxiety is characterized by the presence of disproportionate fear and anxiety in the face of social situations or perceived evaluation (American Psychiatric Association, 2023). Here, the individual is considered a being which is particularly vulnerable to social contact, presenting both a symptomatic dimension and a lived experience that reflects this fragility from an anxious perspective. Articles 4, 16, 17, 18, 19, 20, and 21 focus on the phenomenon of social anxiety beyond a diagnostic category.

In the collected articles, illness in cases of social anxiety unfolds in the dimensions of Time (article 9), Body (article 16, 20), Space (article 4, 18) and the Other (article 19, 21). While alterations in these experiential categories appear in the face of anxious experiences in general, the authors of this category point to the avoidance of social contact as the axis that directs the entire experience of the subject with social anxiety. In this way, the constriction of lived relationships occurs as a way of directing the individual away from vital contact with others, which is seen as aversive and invasive. The author of article 17 states, in relation to lived time, that the individual experiences a constriction and “dysrhythmia” of relational time, shared with others, in a way that directs him to a “social death.” Although the other articles do not use the same terminology, it is clear that the idea of an intentionality that seeks to eliminate the presence of the anxious individual in the face of social contact echoes through the other articles in this category.

While social anxiety presents itself as a form of illness marked by a relational character, productions about the experience of OCD situate it as an experience marked by the insecurity of a subject’s tacit beliefs and meanings in relation to the world. In this way, articles 5, 9, 10, 11, and 19 seek to understand the lived experience of OCD in its relation to anxiety.

As a starting point, it is necessary to understand what the conceptions of the phenomenological literature are regarding OCD. Using Jaspers’ descriptive phenomenology, study 5, by Bürgy (2019), focuses on situating OCD in its descriptive, genetic, and hermeneutical dimensions. For him, the relationship between obsessive thoughts and compensatory compulsive symptoms is still present in the picture. However, beyond a quantitative deviation from common thoughts, it is understood that obsessive thoughts appear as a function of a baseline experience of depersonalization, which manifests itself through a constant feeling of incompleteness. However, the feeling of incompleteness in OCD does not refer to the actual completion of a ritual or task, but to the way the individual defends themself from what is unpredictable. This is a point complemented by the authors of article 9, who situate obsession as a way of dealing with anxiety and anguish, which appear here as the inherent lack of meaning in life.

As previously mentioned, temporality in social anxiety can be described by its relational constriction (Kristiansen, 2023b). In OCD, time is likewise restricted to an infinite present, but for a different reason: the past and the future are avoided because they are uncontrollable, and repetition substitutes for temporal flow (Bürgy, 2019; Doerr-Zegers, 2018). What seems to differ is the target of the uncertainty in the different anxious experiences, as individuals with social anxiety describe themselves as almost dominated by others (Kristiansen, 2023b), whilst the obsessive experience is seemingly marked by an uncertainty of experience in itself.

This understanding is in agreement with what is stated in article 11, by Demaria et al. (2023), which points out the lack of an eidetic fluidity of thoughts and affects in the obsessive experience. For the authors, this obstacle could also hinder the formation of a sense of self that is secure in the ability of one’s own experience to serve as a benchmark for situating oneself in the world. This experience would be strongly linked to a feeling of unfamiliarity with the inhabited world, leading to a spatial, temporal, and relational experience that is not very fluid (Demaria et al., 2023; Esposito and Goretti, 2022). This loss of familiarity, which seems to impede embodiment to be a medium of spontaneous engagement with the world, shows some similarities to the self described bodily and spatial experience in social anxiety (Kristiansen, 2023a; Kristiansen, 2024), though these existential frameworks show different manners of exhibiting this “uninhabitedness.”

In this sense, compulsive behavior would have a compensatory function of trying to establish closure for experiences, but which may eventually fall into repetition. A common understanding then emerges: OCD can be thought of as a lived experience focused, beyond obsessions and compulsions, on repetition as a way of dealing with an unfamiliar temporality and space. In this vein, article 9, by Doerr-Zegers (2018), positions the obsessive lived experience as one marked by anxious time and spatiality, situating phobic and anxious experiences as part of the pathological experience. These studies offer a possible explanatory pathway for comprehension of distinct symptomatic patterns, without abandoning the hypothesis of a shared existential vulnerability in regards to anxious frameworks. Though a majority of works around OCD are theoretical, more empirical studies in Phenomenological Psychopathology may give pathways towards elaborating on how these experiences differ structurally at the level of lived experience.

#### 3.1.3 Studies in clinical practice

In this category, we have gathered articles (2, 8, 13, 15, 22, 25, 26) that offer multiple ways of understanding anxiety, but which, in general, maintain clinical practice as their horizon. Whether deriving reflections from consultations or proposing conceptual or methodological foundations, they all point to implications in the field of care. Phenomenology appears sometimes as a methodological tool for investigation, sometimes as a reference for therapeutic intervention, and sometimes as a basis for the construction of psychopathological concepts of anxiety.

In article 22, by Lu et al. (2024), anxiety is conceived as a transdiagnostic phenomenon, with emphasis on alterations in the temporality and spatiality of the anxious experience. This proximity between theory and clinical practice translates into the development of the Scale for Time and Space Experience in Anxiety (STEA) instrument, whose development and significance are discussed in the article. A clinical trial was conducted on 48 patients diagnosed with anxiety, resulting in convergent validation with that of consistently validated anxiety assessment scales. Here, phenomenological psychopathology concepts are tested on an experimental study outline. The study reinforces a clinical and academic position regarding the validity of a parameterized investigation of the first-person experience of illness, beyond a symptomatic assessment.

Study 8, by Heggdal et al. (2022), introduces the notion of Existential Catastrophe Anxiety (ECA), the result of two decades of empirical research. Phenomenology is present, above all, as a method of accessing patients’ experiences, allowing the description of structuring components of ECA - such as experiential avoidance and dissociation; existential loneliness; undifferentiated emotions and arousal; fear of affective arousal and cognitive avoidance set. Despite the absence of greater phenomenological conceptual depth, the study stands out for the clinical value of the detailed description of experiences linked to contexts of patients living with severe suffering. In studies 15 and 25, by Huguelet (2014) and Temple and Gall (2018) respectively, phenomenology as a philosophical field is integrated into the field of existential psychotherapy. Anxiety is situated as a fundamental condition of existence, in articulation with the themes of death, isolation, meaning, and freedom. Although they do not constitute strictly psychopathological discussions, these works utilized phenomenology in a clinical setting, and whose intervention supported the subjects in the process of sense-making around the subject of anxiety. Study 15 uses anxiety as an illustration of the central challenges addressed in existential psychotherapy, while study 25 uses it as a guiding thread to exemplify the psychotherapeutic process, taking phenomenology as a clinical method and conceptual resource to illuminate the understanding of the experience of the illness.

Study 2, by Bader (2020), attempts to explore anxiety through the concept of social attention. The phenomenological lens, in this case, serves as a theoretical ground that illuminates the intersubjective experience of being-with-the-other, showing how alterations in this domain reverberate in conditions such as social anxiety and borderline personality disorder. The clinical contribution lies in demonstrating that anxiety is not restricted to individual experience, but is expressed in relational dynamics that traverse the field of attention. Like another empirical study, that of study 26, Woodgate et al. (2021) approach clinical practice by investigating the anxiety of young people through metaphors narrated in the first person. Although it does not rely on a robust psychopathological foundation, it offers valuable clinical material by revealing anxiety as a multidimensional experience—bodily, temporal, spatial, and relational. The emphasis on the voice of young people contributes to a sensitive clinical practice.

Study 13, by Glas (2020), presents a theoretical-clinical proposal by articulating phenomenology with the enactive paradigm. For the author, anxiety is not just a response to threats, but a self-referential phenomenon that reveals constitutive aspects of the person themselves. This reading reinforces the intersubjective dimension of symptoms and opens paths for a clinical approach that, beyond the categorical logic of disorders, understands anxiety in terms of vulnerabilities and personal meanings.

Together, the studies point to different paths for the clinical field: one seeks to translate descriptions into applicable instruments (22), others emphasize listening to narratives in contexts of severe suffering (6), or even highlight the therapeutic relevance of the existential perspective (15 and 25). There are those that broaden clinical understanding by including intersubjective dimensions (2) or the first-person voice of specific populations (26). The enactive proposal (13) adds to this picture by articulating phenomenology, science, philosophy and clinical practice. Although they start from different reference points, all the works suggest that phenomenology, in its different modalities, constitutes an indispensable resource for the clinical care of anxiety — whether as a description, method, therapeutic guide, or foundation for new practices.

### 3.2 Anxiety and existential perspectives

The attribution of anxiety as a phenomenon of human nature is a common result among phenomenological productions. These articles (1 to 10), for the most part, position anxiety in contact with concepts of hermeneutic and existential phenomenology, to the detriment of a psychopathological perspective on anxious phenomena.

The Heideggerian conception of anxiety, as well as his elaborations on the concept of “humors,” which speak of different ways of being-in-the-world, constitutes the most common axis of the articles in this category. Articles 1, 4, 5, and 10 align with these conceptions by promoting discussions about anxiety as an experience and ontological axis of Being. Here, anxiety is not conceived as a feeling, but as the relationship between Being and its own incompleteness in relation to the world. The authors mentioned tend to agree that the anxious experience is related to a direct confrontation with the incompleteness of Being; the questioning of one’s own existence, in this case, is directly linked to becoming aware of one’s own possibilities.

In this sense, article 1, by Berenskötter (2020), positions a return to Heidegger as a possibility in the face of the difficulties of experiencing anxiety. For him, anxiety constitutes a paradoxical experience, in which one seeks freedom of action to the same extent that it becomes difficult to appreciate it. Then, the author argues, anxiety is a state wherein a person is confronted by the entire span of their freedom of being; therefore, it is also the way through which one can reinvent itself through an appropriation of their own finitude (based on Heidegger’s concept of *kairós*). Regarding this sequential paralysis and movement through anxiety, the elaborations of Vaškovic and Vičanová (2024) could show a path towards a clinical vision of Heideggerian anxiety, as the authors argue a dialogical relationship between existential anxiety and the axes of experience (Body, Time, Space and Otherness). The article connects them as follows: Death anxiety could reveal an uncertain relationship with Time; Freedom anxiety, with the Body; Meaninglessness anxiety, with Space; and Loneliness anxiety, with Otherness.

Moving away from the Heideggerian perspective, article 10, by Molchanov (2022), proposes a perspective on anxiety and fear based on an approach to Husserl’s concept of phenomenological reduction (or epoché). For the author, the source of the phenomenon of anxiety would originate from contact with otherness, in which contact between the various human “worlds” results in the neutralization of the natural meaning of the world. In this sense, it is a worldly phenomenon that resembles characteristics of epoché, by enabling a conscious reflection on the position that an individual takes on himself and on his world.

There are other articles that use authors typically associated with existentialist schools of philosophy to discuss anxiety. Articles 2 and 3, by Bradley (2015a, 2015b), position understandings based on Friedrich Nietzsche (1844–1900) and Søren Kierkegaard (1813–1855), respectively, in discussions that relate anxiety to the concepts of guilt and social duty. Like the aforementioned article, these articles converge in situating anxiety as an axiomatic experience of encountering otherness, and that guilt in the face of social obligations and internalized forms of duty are a result of this confrontation.

The constitutive relationship between Being and world that makes up the phenomenon of anxiety throughout this category is firmly situated on ontologic or genealogic elaborations, which echo the philosophical fundaments that inform these authors. Establishing a clinical perspective through philosophy, then, would require comprehending how broader concepts such as “freedom,” “guilt” and “duty” come into play in the lived experience of anxious subjects. Though both are theoretical studies, the elaborations of Vaškovic and Vičanová (2024) and Temple and Gall (2018) in studies 24 and 25 of Table 1 stand out for their consideration of a psychopathological anxiety in dialogue with notions of philosophical phenomenology. The articles address anxious experiences that are not part of specific diagnostic categories, which they call “ecological anxiety” and “existential anxiety” respectively, in order to highlight the fragilities that constitute them, and bridging the gap between transcendental concepts and what seems to be the lived experience of anxiety. It could be that a fruitful dialogue between clinical phenomenology and philosophy necessitates a continuous exchange between conceptualization and phenomenological reduction towards understanding the lived experience in its most distinct levels. Though the scope of this review does not allow a weighted evaluation on these contributions, we understand that productions with a clinical direction may strongly benefit from a dialogue with contributions from philosophical phenomenology.

**Table 1:** Main characteristics of the selected articles.

N°	Title	Study type	Journal	References
1	Temporal experience in anxiety: embodiment, selfhood, and the collapse of meaning	Theoretical study	Phenomenology and the Cognitive Sciences	Aho (2020)
2	The human extended socio-attentional field and its impairment in borderline personality disorder and in social anxiety disorder	Theoretical study	Phenomenology and the Cognitive Sciences	Bader (2020)
3	Filling the Silence: Self-Experience and Communication in Social Anxiety	Theoretical study	Erkenntnis	Bortolan (2025)
4	The (un)habitual geographies of Social Anxiety Disorder	Qualitative empirical study	Social Sciences & Medicine	Boyle (2019)
5	Phenomenology of Obsessive-Compulsive Disorder: A Methodologically Structured Overview	Theoretical study	Psychopathology	Bürgy (2019)
6	Exploring the phenomenological structure of existential anxiety as lived through transformative life experiences	Qualitative empirical study	Anxiety, Stress & Coping	Davidov and Russo-Netzer (2022)
7	Clinical Implications of Phenomenological Psychopathology Reflections on a Study of Social Anxiety Disorder	Theoretical study	Philosophy, Psychiatry and Psychology	Fernandez (2025)
8	“Existential Catastrophe Anxiety”: Phenomenology of Fearful Emotions in a Subset of Service Users	Qualitative empirical study	Frontiers in Psychology	Heggdal et al. (2022)
9	Space and Time in the Obsessive-Compulsive Phenomenon	Theoretical study, with a clinical case study as a starting point.	Psychopathology	Doerr-Zegers (2018)
10	Decomposition: Partition as an Escape from Decay - A Gestalt of the Obsessive Life-World	Theoretical study	Psychopathology	Esposito and Goretti (2022)
11	Phenomenological Considerations of the World of the Obsessive Patient	Theoretical study	Journal of Clinical Medicine	Demaria et al. (2023)
12	Obsessive-compulsive existential type: a dialectical-phenomenological approach	Theoretical study	Frontiers in Psychology	Fukuda et al. (2023)
13	An enactive approach to anxiety and anxiety disorders	Theoretical study	Philosophy, Psychiatry & Psychology	Glas (2020)
14	Two ways of combining philosophy and psychopathology of time experiences	Theoretical study	Phenomenology and the Cognitive Sciences	Holzhey-Kunz (2020)
15	The contribution of existential phenomenology in the recovery-oriented care of patients with severe mental disorders	Theoretical study	Journal of Medicine & Philosophy	Huguelet (2014)
16	Forcing an Effortless Stance: The Lived Body in Social Anxiety Disorder	Qualitative empirical study	Psychopathology	Kristiansen (2023a)
17	Struggling for a tomorrow: lived time in social anxiety disorder	Qualitative empirical study	Phenomenology and the Cognitive Sciences	Kristiansen (2023b)
18	Looking for places to be alone: Lived space in social anxiety disorder	Qualitative empirical study	Theory & Psychology	Kristiansen (2024)
19	Feeling like a Perpetual Outsider: Relationality in Social Anxiety Disorder	Qualitative empirical study	Philosophy, Psychiatry & Psychology	Kristiansen (2025a)
20	Revisiting the Phenomenology of Social Anxiety Disorder Lived Body, Gestalt, and Clinical Significance	Theoretical study	Philosophy, Psychiatry & Psychology	Kristiansen (2025b)
21	Expanding the Phenomenology of Social Anxiety Disorder: Loneliness, Absence, and Bodily Doubt	Theoretical study	Philosophy, Psychiatry & Psychology	Krueger et al. (2025)
22	Scale for time and space experience in anxiety (STEA): Phenomenology and its clinical relevance	Construction of a clinical instrument	Journal of Affective Disorders	Lu et al. (2024)
23	Temporal experience as a core quality in mental disorders	Theoretical study	Phenomenology and the Cognitive Sciences	Moskalewicz and Schwartz (2020)
24	Anxiety, Hope and Meaning in Times of Ecological Crisis: An Existential-Phenomenological Perspective	Theoretical study	Human Studies	Vaškovic and Vičanová (2024)
25	Working Through Existential Anxiety Toward Authenticity: A Spiritual Journey of Meaning Making	Theoretical study	Journal of Humanistic Psychology	Temple and Gall (2018)
26	Understanding Youth’s Lived Experience of Anxiety through Metaphors: A Qualitative, Arts-Based Study	Qualitative empirical study	Int J Environ Res Public Health	Woodgate et al. (2021)

## 4 Discussion

It is clear from this review that there is a pluralistic discussion about the phenomenon of anxiety within the scope of phenomenology. Productions related to phenomenological psychopathology stand out, as they can serve as a starting point for a critical understanding of the experience of the illness, without reducing it to anxious symptomatology and presenting clues for clinical interventions. In the exercise of describing the alterations in the experience of being in an anxious world, authors in the field position anxiety as a global phenomenon that transcends (but is also present in) psychopathological conditions (Lu et al., 2024; Glas, 2020; Huguelet, 2014).

The description of the singular and subjective experience, which is the basis of phenomenological psychopathology, points to the presence of existential categories that position the ways in which an anxious individual situates themself in the world. Among these conditions, disturbances in the experience of time are most frequently associated with anxiety (Aho, 2020; Kristiansen, 2023b; Holzhey-Kunz, 2020; Moskalewicz and Schwartz, 2020). This may be related to the fact that, historically, temporality has been the most investigated phenomenological category in experiences of illness in the field of phenomenological psychopathology (Minkowski, 1933/1995; Tatossian, 1979/2006). However, it is important to note that disturbances in temporality are not exclusive to anxiety. Rather, what distinguishes anxious temporality seems to be an anticipatory dread related to the future horizon, described in such terms as a dysrhythmia (Aho, 2020), acceleration (Moskalewicz and Schwartz, 2020), and narrowing of the temporal horizon (Kristiansen, 2023b).

Phenomenological literature emphasizes that the experience of the world depends on the ability to retrieve the past and project oneself into the future, which enables the individual’s positioning in the present (de Souza et al., 2020; Minkowski, 1933/1995). In this context, anxious temporality hinders the healthy flow of time, with aversive sensations not located in the past or future, but trapped in an eternal present (Kristiansen, 2023b; Aho, 2020).

It is important to emphasize that, although the extent of alterations in temporality is discussed, and people who present them may be more sensitive to anxious experiences (Holzhey-Kunz, 2020), the collected articles do not consider a causal relationship between these phenomena. Anxiety and temporality disorders are mutually constitutive, as they are expressions of an individual’s lived world, making it difficult to pinpoint the cause of the anxious state based on these phenomena in isolation. However, it is understood that investigations into the origin of the anxious experience as a global alteration of subjectivity can be of great clinical and academic benefit, and without these investigations there would be a possible gap in the literature (Table 2).

**Table 2:** Main characteristics of the selected articles.

N°	Title	Key theorists	Journal	References
1	Anxiety, time, and agency	Heidegger & Kierkegaard	International Theory	Berenskötter (2020)
2	Brokenness and hope: David Lynch’s contribution to a phenomenology of anxiety in Mulholland Drive	Nietzsche	The Journal of Speculative Philosophy	Bradley (2015a)
3	Ligatio ex Nihilo: Original sin and the hope for redemption: An alternate path through the darkness	Kierkegaard	International Philosophical Quaterly	Bradley (2015b)
4	Phenomenological yields of anguish in Heidegger	Heidegger	Alpha - Revista de Artes, Letras y Filosofia	Chillon (2018)
5	Anxiety and Fear as Moods of Everydayness in Heidegger’s Being and Time	Heidegger	Problemos	Grigas (2022)
6	Paul Ricoeur on the recognition of anxiety: Phenomenological hermeneutics in action	Heidegger, Kierkegaard & Ricoeur	RUDN Journal of Philosophy	Innokentievna (2021)
7	Being and Time-less Faith: Juxtaposing Heideggerian Anxiety and Religious Experience	Heidegger	Open Theology	Lyonhart (2020)
8	The Nothing of Mallarmé with the Anxiety of Heidegger	Heidegger & Mallarmé	Modern Theology	Marion (2024)
9	Dis-chronic Experience of No-thing: Existential Analysis of Freud’s and Heidegger’s Concept of Anxiety	Heidegger & Freud	Eidos	Mauri (2020)
10	Epoche and Anxiety. Neutralization of the World or Imitation of the Experience?	Husserl	Horizon	Molchanov (2022)

It should be noted that the phenomenological literature is not restricted to the category of time in understanding anxiety. Other studies broaden the debate by highlighting effects on spatiality (Kristiansen, 2024; Lu et al., 2024; Boyle, 2019), as well as on the relationship with the body and with others (Kristiansen, 2023a; Kristiansen, 2025a; Boyle, 2019). From this perspective, anxiety is understood as a disturbance in the way of inhabiting the world, in which the anxious individual simultaneously experiences the invasion of spaces and relationships and the feeling of being invaded by them, especially by the demands of others (Boyle, 2019; Kristiansen, 2023a; Kristiansen, 2024; Lu et al., 2024).

This understanding of anxiously inhabiting the world allows for a dialogue with nosographic formulations. Authors such as Boyle (2019) and Glas (2020) describe anxiety as a set of experiences marked by the anticipation and avoidance of aversive contact. However, by deepening its self-referential dimension, they indicate that anxiety is not reduced to an isolated affective state, but is configured as a learned way of situating oneself in the world. Thus, the anxious individual tends to constitute a field of meanings in which anxiety operates as a structuring perspective of experience, approaching, albeit implicitly, the phenomenological notion of an anxious lived world.

This discussion finds particular resonance in articles that discuss the experience of social anxiety. Although the articles analyzed do not suspend the diagnostic definitions of this condition, phenomenology allows us to understand it as a direction of lived experience towards one’s own behavior in the intersubjective encounter, guided by the constant attempt to anticipate, satisfy, or avoid the expectations of others (Boyle, 2019; Kristiansen, 2023b). In this context, contact with the other acquires an overwhelming character, so that fears and attitudes become primarily organized according to the presence of others, to the detriment of the very possibility of inhabiting the world. This is, therefore, an existential posture marked by instrumentalization and, in certain cases, by the annihilation of oneself in favor of the other (Kristiansen, 2023a, 2023b, 2024). The intensification of self-awareness and self-criticism, although it may broaden the perceptive field, sustains a persistent anxiety that makes more unreflective ways of being-in-the-world unfeasible (Boyle, 2019; Bader, 2020; Kristiansen, 2023b).

It must be noted here that what appears to distinguish anxiety is not the presence of alterations in phenomenological structures, but their specific configuration: an acceleration and narrowing of the future horizon associated with an imminent threat, alongside a hypervigilance regarding the social field (Kristiansen, 2023a; Boyle, 2019). It is through this composition of experiences that anxiety distinguishes itself from other psychopathological experiences that share fragilities in structural temporality, spatiality, embodiment and intersubjectivity, such as depression or schizophrenia. However, the present review does not allow for a definitive statement as to whether any single feature or signal could be exclusive to clinical anxiety. As such, comparative studies across disorders are required to determine this.

It is from this understanding of anxiety as a fragile mode of relating to the world that the focus on articles dedicated to the phenomenology of obsessive-compulsive disorder (OCD) is justified. Beyond its historicity in phenomenological studies, recent literature points to important convergences between these fields. Authors such as Fukuda et al. (2023) and Esposito and Goretti (2022) see OCD as a specific “existential type,” describing the obsessive experience as marked by a radical difficulty in habituation to the world, which is expressed in the rigidity of self-imposed rules and meanings. More than the articulation between obsessive thoughts and compulsive behaviors, it is the fundamental uncertainty in the relationship with reality that weakens the obsessive subject (Bürgy, 2019; Demaria et al., 2023). This fragility, according to Esposito and Goretti (2022), favors the creation of rigid normative systems as an attempt to stabilize the lived world, even if supported by untruths or harmful existential patterns (Demaria et al., 2023).

The different anxiety disorders reviewed share an existential fragility similar to that of OCD, but this fragility is constituted with distinct meanings in each condition. In panic and generalized anxiety, the disturbance manifests primarily through a diffuse openness to the world, in which uncertainty saturates the experiential field and remains dispersed and non-localized (Doerr-Zegers, 2018; Moskalewicz and Schwartz, 2020). In OCD, this same constitutive uncertainty is confronted through a radical narrowing of the intentional field. The obsessive subject loses distance from the world by fixating on a single theme or act, from which they cannot detach themselves (Denys, 2011).

This fixation appears to correspond to a basal experience of depersonalization, marked by a constant feeling of incompleteness and estrangement from the inhabited world. Obsessive thoughts emerge as a response to this uncertainty, while compulsive behavior fulfills a compensatory function, seeking to produce closure for such experiences. This closure tends to fall back into repetition, which evidences the ineffective and provisional character of this circumscription (Denys, 2011). The obsessive quest for certainty thus reveals a basal distrust in the subject’s relationship with the world and with themselves.

de Haan et al. (2013) deepen this condition by situating it in a pre-reflective order. This uncertainty reverberates in a quest for control, exercised through an excess of reflective awareness. The patient begins to deliberately attend to what should occur spontaneously and unthematized. This hyperreflexivity structurally distinguishes OCD from the other anxiety disorders reviewed. Classic anxiety disorders preserve, albeit in a weakened form, the pre-reflective structure of experience, whereas OCD replaces it with a regime of conscious vigilance. This regime, paradoxically, deepens the very fragility it sought to remedy (de Haan et al., 2013). This differentiation offers a possible explanatory pathway for the genesis of distinct symptomatic patterns among these conditions, without abandoning the hypothesis of a shared existential vulnerability.

Beyond understanding anxiety as an affective signal present in OCD (American Psychiatric Association, 2023), the phenomenological literature allows us to establish a direct proximity between the anxious lived-world and the obsessive lived-world. Although they are distinct psychopathological phenomena, both configure ways of being-in-the-world marked by the loss of naturalness in the relationship with reality. From this perspective, a constriction of the field of meanings is evident, in which uncertainty, experienced as a threat, limits experiential openness and hinders the constitution of new meanings (Doerr-Zegers, 2018; Demaria et al., 2023; Moskalewicz and Schwartz, 2020). Still, the contemporary production analyzed does little to explore the genesis of the symptomatic differences between classic anxiety disorders and OCD, despite suggesting the presence of similar existential vulnerabilities. In this sense, anxiety emerges as a transversal phenomenon, simultaneously mundane and psychopathological, present in different clinical pictures, even in the absence of shared diagnostic criteria.

In discussions of a primarily philosophical nature, the concept of anxiety is seen as a human and existential phenomenon, and the discussions elaborated tend to revisit classic authors of phenomenology and existentialism, such as Martin Heidegger, Jean-Paul Sartre, and Søren Kierkegaard. This is largely due to an epistemic problem: the confluence between terms from modern philosophy, such as “angst” and “anguish,” and those used by academics in Psychology, such as “fear” and “anxiety,” to signify the same phenomenon (Micali and Mickiewicz, 2023; Micali, 2022). The presence of these authors in debates in Psychology, even though they do not explicitly discuss clinical practice, strengthens a view of anxiety as a human phenomenon that is implicitly related to these concepts. Phenomenological clinical practice, in its exploration and support of lived anxiety, is likely to encounter a strong existential axis of anxiety, so that an approximation between clinical practice and philosophy proposes a more complete practice in both fields.

The distribution of collected articles appears to prioritize fields that, at first glance, are antagonistic in their conception of anxiety. While authors of phenomenological psychopathology tend to understand the experience of anxiety as that related to the avoidance of one’s own experience and the world (Aho, 2020; Boyle, 2019; Kristiansen, 2023a; Kristiansen, 2023b; Glas, 2020), productions of philosophical phenomenology, especially those aligned with Heideggerian thought, tend to position anxiety (and anguish) in relation to the possibility of centering one’s gaze on one’s own experience (Berenskötter, 2020; Grigas, 2022; Mauri, 2020; Molchanov, 2022). The distinctness of these may be understood to come from a difference in the level in which the phenomenon of anxiety being described. While a psychopathological perspective may understand it as an experience in the *ontic* level, as it is lived by a subject in the world, the philosophical tradition attempts to describe it as an *ontological* structure of being in the world. It is assumed here that these are not opposing views; clinical phenomenology may utilize an ontological comprehension for its practice while paying close attention to the concrete suffering of its subject. However, this is a reading that stems from an attempt at bridging these disciplines, and is not outright established in these papers with the exception of Holzhey-Kunz (2020). As such, the authors of this article suggest careful interpretation of the meaning of anxiety as it appears in distinct fields of research.

From the perspective of philosophical phenomenology, anguish appears as a phenomenon synonymous with anxiety, even though it is understood that the idea of a conceptual “synonymity” is not faithful to phenomenological investigation (Micali and Mickiewicz, 2023). The ways of accessing anguish are multiple: encountering uncertainty (Berenskötter, 2020), otherness and the demands of Others (Bradley, 2015a, 2015b; Molchanov, 2022), or the incompleteness of experience (Chillon, 2018; Mauri, 2020; Marion, 2024; Lyonhart, 2020). However, inseparable from these conclusions lies a common direction for the cases of anxiety portrayed in these articles. Through anguish, and a direct confrontation with human incompleteness, the anxious individual can move towards a creative production of meaning. Although encountering anxiety and anguish removes the individual from the comfort of tacit senses, it also allows them to question the meaning of their own life and potentially take a stand on their choices.

This direction resonates in productions related to the clinical aspects of phenomenology. Bearing in mind clinical contact as a means of exploration, anxiety can be understood from its lived meaning, in the description of sensations found in everyday life but which haunt the individual in treatment (Temple and Gall, 2018; Woodgate et al., 2021). Anxiety, both in psychopathological conditions and in broader senses, tends to be related to existential fragilities that are revealed in clinical contact; death, freedom, the judgment of others, loneliness, etc., these speak of fears of being affected by the contents of life (Heggdal et al., 2022; Davidov and Russo-Netzer, 2022).

It is in this sense that the clinical approach to this mundane anxiety becomes evident. The reviewed literature suggests that phenomenologically read clinical practice offers a framework for exploring the lived experience in anxiety, especially when it seems to lack tacit meaning. Several authors propose that this exploration may potentially support the emergence of alternative, original meanings to that experience through clinical contact (Woodgate et al., 2021; Demaria et al., 2023).

## 5 Final considerations

Through this study, we have highlighted the manner in which different fields that use a phenomenological lens examine anxiety. Although the discussions are distributed across areas of knowledge with distinct specificities, the focus on clinical care as an instrument for sense-making is highlighted, even (or especially) in the face of uncertainty. The results obtained from the data collection also highlight a plurality of findings regarding the phenomenon of anxiety, in its affective, psychopathological, and existential expressions.

Philosophical discussions about anxiety are discussed in their respect towards existential notions of anguish, uncertainty, and the incompleteness of life, situating them in contemporary times and allowing for comprehensive depth in the face of such a complex phenomenon. Although they do not produce a psychological perspective on anxiety, this discussion is understood as fruitful for a reflection on clinical practice as a form of investigation of the lived experience itself. In the context of a growing number of anxiety diagnoses, it is necessary for clinical psychology, equipped with current tools and understandings, to critically address human anguish, seeking to understand its mundane and pathological aspects.

While the findings of this article offer contributions to clinical and health psychology, a majority of the data collected is conceptual or qualitative-descriptive, and establishing a positive relation between certain interventions and standardizable treatment for anxiety was not in the scope of this research. By mapping the different ways in which anxiety has been phenomenologically understood, the study may provide clinical psychologists and healthcare professionals with an expanded repertoire for interpreting anxious suffering beyond conventional diagnostic criteria. Future experimental research is necessary to evaluate the implications of these concepts in practice.

The data collected through this review point to contemporary advances in the investigation and direction of treatment for anxiety and associated disorders. However, the absence of studies that engage with phenomenological conceptions of anxiety in relation to factors concerning contemporary life, such as exhausting work relationships, communication technologies and digital media, intersectionalities of gender, race and social class, as well as the phases of human development, such as childhood, adolescence and old age, is notable. This is understood as a gap in research, as these are themes of paramount importance for a critical and situated understanding of the experience of the illness today, and which inevitably permeate the clinical setting.

## Appendix

As previously mentioned, database-specific searching tools restricted the usage of the optimized search string. The following is a list of adaptations to the original query. All articles collected this way were filtered by a duplicate detection mechanism on Rayyan (Ouzzani et al., 2016) following their collection.

### BVP Psi

Utilizing the “advanced research” tool, filling every search box with an appropriate descriptor and boolean operator, and tuning it to search for “title, abstract, subject”, the following string was obtained:

(phenomenology) OR (phenomenological) OR (phenomenological approach) OR (phenomenological analysis) OR (lived experience) OR (existential perspective) AND (anxiety) OR (anguish) OR (angst) OR (fear) OR (generalized anxiety disorder) OR (social phobia) OR (specific phobias) OR (separation anxiety disorder) OR (selective mutism) OR (substance/medication-induced anxiety disorder) OR (anxiety disorder due to a medical condition) OR (obsessive-compulsive disorder) OR (OCD)

### Scopus

Utilizing the “advanced search” tool, and due to a restriction in boolean operators, the following strings were utilized:

(“phenomenolog*” OR “lived experience” OR “existential perspective”) AND (“anxiety” OR “anguish” OR “angst” OR “fear” OR “generalized anxiety disorder” OR “panic disorder”)

(“phenomenolog*” OR “lived experience” OR “existential perspective”) AND (“social anxiety disorder” OR “social phobia” OR “specific phobias” OR “separation anxiety disorder” OR “selective mutism” OR “substance/medication-induced anxiety disorder”)

(“phenomenolog*” OR “lived experience” OR “existential perspective”) AND (“substance/medication-induced anxiety disorder” OR “anxiety disorder due to a medical condition” OR “obsessive-compulsive disorder” OR “OCD”)

### Web of Science

Utilizing the “advanced research” tool, the following string was utilized:

(“phenomenology” OR “phenomenological” OR “phenomenological approach” OR “phenomenological analysis” OR “lived experience” OR “existential perspective”) AND (“anxiety” OR “anguish” OR “angst” OR “fear” OR “generalized anxiety disorder” OR “GAD” OR “panic disorder” OR “social anxiety disorder” OR “social phobia” OR “specific phobias” OR “separation anxiety disorder” OR “selective mutism” OR “substance/medication-induced anxiety disorder” OR “anxiety disorder due to a medical condition” OR “obsessive-compulsive disorder” OR “OCD”)

### PubMed

Utilizing the “advanced research” tool, and tuning the search for “all fields,” the following string was obtained:

(“phenomenology” OR “phenomenological” OR “phenomenological approach” OR “phenomenological analysis” OR “lived experience” OR “existential perspective”) AND (“anxiety” OR “anguish” OR “angst” OR “fear” OR “generalized anxiety disorder” OR “GAD” OR “panic disorder” OR “social anxiety disorder” OR “social phobia” OR “specific phobias” OR “separation anxiety disorder” OR “selective mutism” OR “substance/medication-induced anxiety disorder” OR “anxiety disorder due to a medical condition” OR “obsessive-compulsive disorder” OR “OCD”)
